# Neuroinflammation Markers in Tear Fluid of Mild Alzheimer’s Disease

**DOI:** 10.1007/s12031-025-02368-x

**Published:** 2025-06-05

**Authors:** Virve Kärkkäinen, Toni Saari, Minna Rusanen, Hannu Uusitalo, Ville Leinonen, Bernd Thiede, Kai Kaarniranta, Anne M. Koivisto, Tor P. Utheim

**Affiliations:** 1 NeuroCenter, Neurology, Kuopio University Hospital, P.O. Box 100, FI-70029 KYS, Kuopio, Finland; 2 NeuroCenter, Neurosurgery, Kuopio University Hospital, Kuopio, Finland; 3 Neurosurgery, Institute of Clinical Medicine, School of Medicine, University of Eastern Finland, Kuopio, Finland; 4 Institute for Molecular Medicine Finland (FIMM), HiLIFE, University of Helsinki, Helsinki, Finland; 5 Ceriatric Center, Wellbeing Services Country of North Karelia, Joensuu, Finland; 6 Eye and Vision Research, Faculty of Medicine and Health Technology, Tampere University, Tampere, Finland; 7 Department of Biosciences, University of Oslo, Oslo, Norway; 8 Department of Ophthalmology, Institute of Clinical Medicine, School of Medicine, University of Eastern Finland and Kuopio University Hospital, Kuopio, Finland; 9 Department of Molecular Genetics, University of Lodz, Lodz, Poland; 10 Department of Geriatrics, Helsinki University Hospital and Department of Neurosciences, University of Helsinki, Helsinki, Finland; 11 Department of Ophthalmology, Oslo University Hospital, Oslo, Norway; 12 Department of Medical Biochemistry, Oslo University Hospital, Oslo, Norway

**Keywords:** Alzheimer’s disease (AD), Tear fluid (TF), Proteomics, AD pathophysiology, Inflammation, Biomarkers, Koivisto AM and Utheim TP share the last authorship.

## Abstract

The protein composition of tear fluid (TF) reflects the severity and progression of many age-related diseases. Here, we evaluated TF proteins from patients with mild Alzheimer’s disease (AD) and cognitively healthy controls (CO) to explore potential new biomarker molecules. The aim of this study was to explore potential new biomarker molecules by examining the expression of TF proteins whose function is related to neuroinflammation. We examined 53 participants (34 COs, mean age 71 years, Mini-Mental State Examination (MMSE) score 28.9 ± 1.4; 19 with AD, Clinical Dementia Rating 0.5–1, mean age 72 years, MMSE 23.8 ± 2.8). All participants underwent neurological status examination, cognitive testing, and ophthalmological examination. TF was collected using Schirmer strips, and TF protein content was evaluated using mass spectrometry-based proteomics and label-free quantification. We report 14 TF proteins that showed altered protein expression in the AD group compared to the CO group. Twelve proteins were significantly upregulated (SERPINA3, FGA, SIAS, ORM1, ANXA3, G6PI/NLK, CH3L2, MSLN, CPPED1, JCHAIN, IGHV5-51, SPARCL1) and two were downregulated (PIP, SCGB2A1) (*p* ≤ 0.05). Observed altered expression of TF proteins in the AD group may have potential in AD pathology. Since inflammation is one of the earliest signs of neurodegeneration in AD, these proteins are putative new biomarker candidates of early AD.

## Introduction

Alzheimer’s disease (AD) is the most common neurodegenerative disease and cause of dementia globally. It is a multifactorial and progressive disease that ultimately leads to a massive loss of neurons and synapses in the patient’s brain. Pathophysiological changes in the AD brain typically start to develop years before clinical signs of AD occur (Kenny et al. 2019). The diagnosis of AD is usually made relatively late, when the patient has mild memory and behaviour deficits, may need help in everyday activities, and care costs are increasing (Jetsonen et al. 2021).

Despite 25 years of intensive research into Alzheimer’s disease drug development, there is no treatment options which can prevent the progression of the disease. Available medical therapies only alleviate the symptoms and may slightly slow down the progression of AD (Rajendran and Krishnan 2024). Drugs for early and mild Alzheimer’s disease that have recently entered clinical use in some countries are targeted on the clearance mechanisms of amyloid plaques (Nalivaeva and Turner 2019) while the other symptomatic therapies alleviate acetylcholine deficiency by cholinesterase inhibitors (Grutzendler and Morris 2001). There are also other therapies under research for AD, e.g. neuroprotective proteins (Fan et al. 2022; Fan et al. 2025). To reduce the burden of AD, we urgently need new treatment options, tests, and biological markers to detect AD earlier and improve diagnostic accuracy. The search for easy-to-use, cheaper, and more specific AD biomarkers and the new sources for them has become an intensively studied topic of research (Kenny et al. 2019; Yaqub et al. 2023; Kalló et al. 2016; Kaštelan et al. 2023).

The best-known hallmarks of the AD brain are the overproduction and extracellular deposition of the amyloid-ß (Aß) peptide and intracellular aggregation of hyperphosphorylated tau (p-tau) protein. Both proteins are highly aggregating and exist to a lesser extent in the healthy brain in these forms (Selkoe 2008; Wang and Mandelkow 2016). These depositions are thought to be initiators for the development of other hallmarks such as oxidative stress, inflammation, and neuronal death (Selkoe 2008; Wang and Mandelkow 2016; Kiraly et al. 2023).

In the healthy brain, microglia and astrocytes support neuronal survival and maintain the homeostasis of synaptic transmission and dendritic pruning (Liddelow et al. 2017). Microglia are the primary immune cells in the brain, with the primary function of phagocytosis of cellular debris and pathogens. They also activate astrocytes to respond to pathogens or disease (Liddelow et al. 2017). In the AD brain, Aβ and p-tau aggregates activate astrocytes and microglia, inducing a reactive state and changing their roles. Unlike in their resting states, the activity of reactive microglia and astrocytes can contribute to the development of inflammation and induce neuronal apoptosis (Kiraly et al. 2023; Liddelow et al. 2017; Pelkmans et al. 2024). The function of reactive glial forms is more complicated, as their activity also contributes to the development of inflammation and induces neuronal apoptosis (Kiraly et al. 2023).

Neuroinflammation has been described as starting when reactive microglia and astrocytes lose their homeostatic state and begin to secrete an inappropriate number of neurotrophic factors and pro-inflammatory cytokines. Simultaneous systemic inflammation causes damage to the blood-brain barrier. Chronic inflammation leads to the activation of the anti-inflammatory system, through which neuroprotective interleukins are produced. In the AD brain, chronic inflammation leads to an increased amount of aggregated tau and Aß-peptides, which has been shown to worsen the effect (Kiraly et al. 2023). In the early stages of AD, reactive astrocytes are reported to surround the Aß plaques and tau deposits and release pro-inflammatory cytokines and chemokines (Pelkmans et al. 2024).

Tear fluid (TF) is protein-rich, consisting of a wide variety of enzymes, neuropeptides, and protective proteins. TF proteins are either secreted from the lacrimal gland or transported to TF from serum (Zhou and Beuerman 2017). TF is considered a potential source of biomarker molecules; recent studies have reported that in systemic diseases such as AD, disease-altered protein expression can be detected in TF samples (Yaqub et al. 2023; Kalló et al. 2016; Król-Grzymała et al. 2022; Gijs et al. 2021). Kalló et al. 2016. reported downregulated expression of the TF proteins lysozyme-C, lactotransferrin, prolactin, lipocalin-1, and lacritin in patients with AD (Kalló et al., 2016). Meanwhile, Gijs et al. have reported the detection of tau and Aß peptides in TF (Gijs et al. 2021).

Our hypothesis was that neuroinflammation may cause changes in TF protein functions. Therefore, alterations in TF proteins may reflect AD progression and serve as potential biomarkers. To compare protein expression levels, we performed proteomic analysis of TF collected from patients with AD (AD group) and cognitively healthy controls (CO group).

## Methods

### Ethics Statement

The study adhered to the principles of the Declaration of Helsinki and was evaluated and accepted by the Research Ethics Committee of the Northern Savo Hospital District (Dnro: 482/2017). All study participants were verbally informed, provided with an information sheet, and signed informed consent prior to participation. Proxy consent was signed for individuals diagnosed with AD.

### Study Design and Study Protocol

For this cross-sectional pilot study, a total of 53 volunteers over 60 years of age were recruited from the Brain Research Unit of the University of Eastern Finland and the memory clinic of Kuopio University Hospital NeuroCenter. Within this group, 19 had been diagnosed with AD before enrolling in this study (AD group), and 34 were considered cognitively healthy and were used as a control group (CO group). AD was diagnosed by a geriatrician or neurologist at the memory clinic before the year of 2018, based on the revised National Institute on Aging and Alzheimer’s Association criteria (McKhann et al. 2011). Before diagnosing AD, other etiologies of memory decline were excluded through brain imaging, comprehensive cognitive testing (Consortium to Establish a Registry for Alzheimer’s Disease (CERAD) neuropsychological battery or by larger neuropsychological tests), and differential diagnostic laboratory tests. AD diagnosis has been supported by biomarker examinations and by monitoring them clinically years after the diagnosis.

The exclusion criteria for this study were memory decline due to etiologies other than AD; limited ability to cooperate due to moderate to severe AD; diabetes; and eye diseases, such as glaucoma, dry-eye disease, or age-related macular degeneration.

At the first study appointment, comprehensive demographic information was obtained from participants and their family members, including medical history, neurological signs and symptoms, medication, and family history of AD (Table 1). All participants were evaluated using the Clinical Dementia Rating (CDR) scale (Morris 1993). Participants with any signs of upper motor neuron compromise, parkinsonism, balance abnormalities, or ataxia were excluded. All participants were evaluated using the Finnish version of the CERAD neuropsychological test battery, including the Mini-Mental State Examination (MMSE) (Hänninen et al. 2010).

**Table 1:** Demographic data of the control (CO) and Alzheimer’s disease (AD) study groups

	CO	AD	*p* (CO-AD)
Number of participants, *n*	34	19	
Age, years (±SD)	71 (5.3)	71 (6.8)	0.817
Gender, female, *n* (%)	19 (56)	12 (63)	0.413
MMSE result, max 30 (±SD)	28.6 (1.4)	23.9 (2.8)	**0.000**
MMSE, range	25–30	16–27	Nap
Family history, *N* (%)	19 (57.6)	12 (63.2)	0.462
BMI, kg/m^2^ (±SD)	25.3 (2.6)	24.5 (3.4)	0.338
Coronary artery disease, *n* (%)	3 (8.8)	2 (10.5)	0.596
Hypercholesterolemia medication, *n* (%)	14 (41.2)	5 (26.3)	0.218
Hypertension medication, *n* (%)	19 (55.9)	12 (63.2)	0.413
ApoE allele 4 carriers, *n* (%)	15 (44.1)	14 (82.4)	**0.009**

Bold indicates significant differences between groups at *p* ≤ 0.05

*CO*, cognitively healthy control group; *AD*, Alzheimer’s disease group; *SD*, standard deviation; *MMSE*, Mini-Mental State Examination (range 0–30); *ApoE*, apolipoprotein E; *Nap*, not applicable

Study participants whose CERAD results were within the normal range and who experienced no decline in daily activities based on demographic information and CDR interviews (CDR0) were considered cognitively healthy controls (CO). Study participants in the AD group represented mild AD dementia; they all had a diagnosis and medication for AD, and they showed a mild decline in daily activities based on the CDR interview (CDR0.5-1.0).

### ApoE Genotyping

Genomic DNA was extracted from venous blood samples using the QIAamp DNA Blood Mini Kit (QIAGEN). *ApoE* alleles were determined using TaqMan genotyping assays (Applied Biosystems (ABI), Foster City, CA, USA) for two single nucleotide polymorphisms (rs429358 and rs7412) and an allelic discrimination method on the ABI 7000 platform (De la Vega et al. 2005).

### Eye Examination

Slit-lamp examination was performed using a CSO biomicroscope (Costruzuzione Strumenti Oftalmici, Firenze, Italy). Tear film stability was assessed based on tear film break-up time (tBUT), and Schirmer test strips were used to measure tear fluid production (Jauhonen et al. 2015; Laihia et al. 2020). Lid margin and bulbar conjunctival redness were scored according to the Institute of Eye Research grading scales (very slight, 0; mild, 1; moderate, 2; or severe, 3) (Jauhonen et al. 2015). Corneal and conjunctival fluorescein staining patterns were scored using the Oxford grading scale, from 1 (absent) to 5 (marked) (Laihia et al. 2020).

### Tear Collection and Sample Preparation

TF samples were collected using Schirmer strips (Tear Touch; Madhu Instruments) without anaesthesia. The strips were placed under the lower eyelid for 5 min, and after removal, placed into 1.5 ml Eppendorf tubes and stored at −80 °C to await further processing.

### In-Solution Protein Digestion

Proteins from the Schirmer strips were eluted with 200 μl PBS before being precipitated with cold acetone. In-solution digestion of the proteins was then performed with 2 μg trypsin GOLD (Promega, Madison, WI, USA) after reductive alkylation using DTT and iodoacetamide.

### Protein Identification and Label-Free Quantification

Tryptic peptides were purified using 10 μl OMIX-C18 micro-SPE pipette tips (Agilent, Santa Clara, CA, USA). Next, the purified peptides were injected into the liquid chromatography/mass spectrometry (LC/MS) system, comprising a timsTOF Pro (Bruker Daltonik, Bremen, Germany) coupled online to a nanoElute nanoflow liquid chromatography system (Bruker Daltonik, Bremen, Germany) via a CaptiveSpray nanoelectrospray ion source. The LC/MS data were searched against the human Uniprot database (20,431 entries), using PEAKS X+ software version 10.5 (Bioinformatics Solutions, Waterloo, ON, Canada). A false-discovery rate (FDR) of 1% was applied to the data sets.

**Fig. 1:**
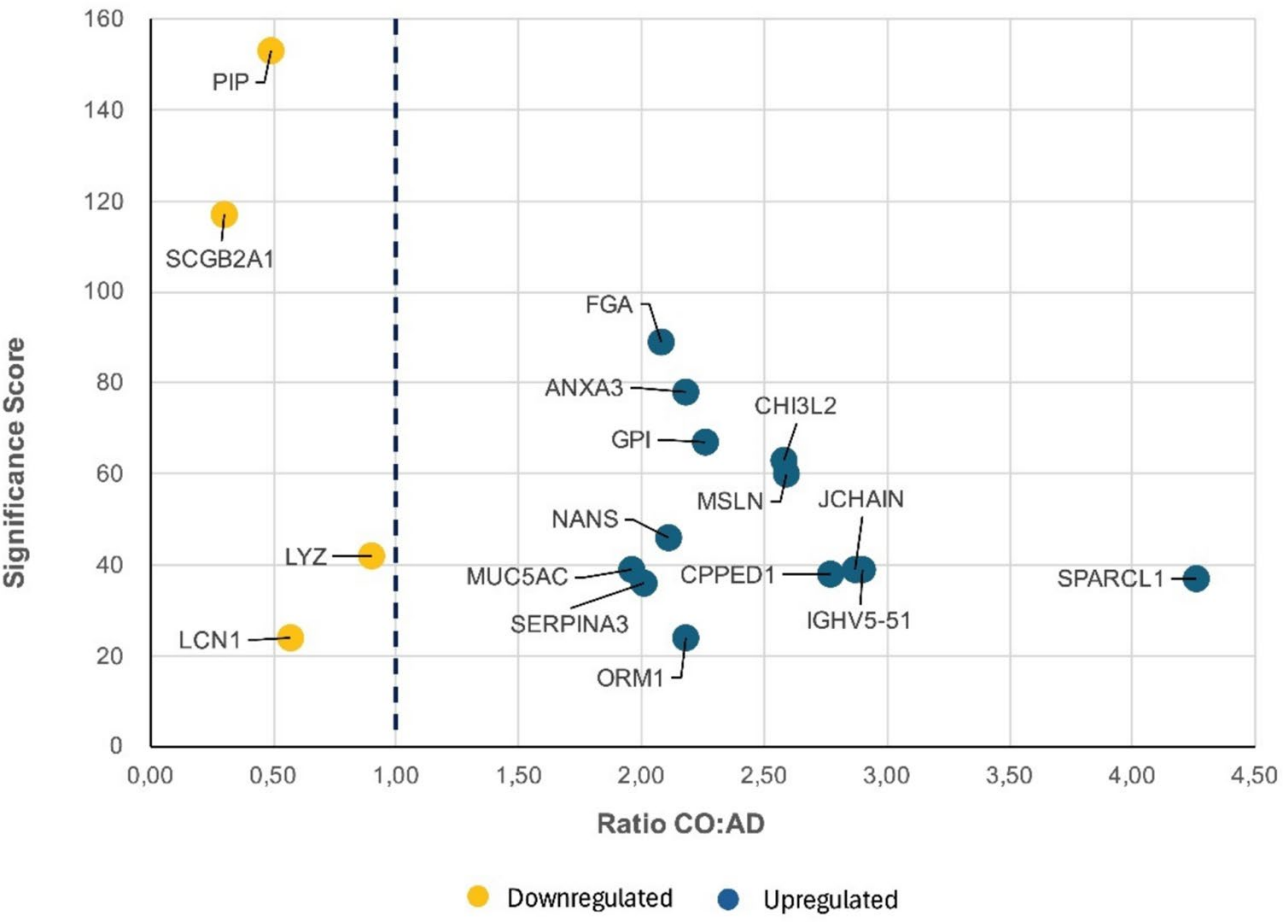
The up- and downregulated tear fluid (TF) proteins

For label-free quantification using PEAKS, ID-directed label-free quantification was performed with outlier removal. The following parameters were applied regarding peptide features: quality ≥ 4, peptide ID count per group ≥ 1, and detected in ≥ 1 sample per group. The following parameters were applied to the proteins: FDR ≤ 5%, fold change ≥ 2, and significance on analysis of variance with ≥ 2 peptides. The significance score was calculated as the −10 log10 of the significance testing *p*-value. Up- and downregulated proteins are presented in Figure 1. The figure was created using Excel.

### Statistical Analysis

Statistical analyses were performed using SPSS 22 software (SPSS Inc., Chicago, IL, USA). A *p-*value of <0.05 was considered a significant result. Results regarding demographic data, CERAD test (MMSE), and ocular data (Tables 1 and 2) are presented as mean ± standard deviation (SD), or as number of cases and proportions. Comparisons were made using *t*-tests and one-way analysis of variance, followed by the least-significant difference post-hoc test. Group differences were expressed as mean difference with 95% confidence intervals.

**Table 2:** Ocular data for control (CO) and Alzheimer’s disease (AD) study groups

Ocular data	CO *n* = 34	AD *n* = 19	*p*-value (CO-AD)	Mean difference (95% confidence interval)
Schirmer, mm in 5 min, right eye	9.28 (6.9)	8.3 (8.2)	*0.648*	*0.96 (−3.53 to 5.45)*
Schirmer, mm in 5 min, left eye	9.03 (6.1)	11.2 (8.3)	*0.281*	*−2.13 (−6.52 to 2.26)*
TBUT, right eye	5.54 (3.1)	5 (3.8)	*0.579*	*0.54 (−1.56 to 2.64)*
tBUT, left eye	5.6 (3.2)	5 (3.8)	*0.545*	*0.6 (−1.51 to 2.71)*
Conjunctival redness, right eye			*0.025*	
0 = no redness, % (*n*)	0	0		
1 = mild redness, % (*n*)	58.3 (21)	85 (17)		
2 = moderate redness, % (*n*)	41.7 (15)	10.0 (2)		
3 = severe redness, % (*n*)	0	5 (1)		
Conjunctival redness, left eye			*0.062*	
0 = no redness, % (*n*)	0	0		
1 = mild redness, % (*n*)	58.3 (21)	80.0 (16)		
2 = moderate redness, % (*n*)	41.7 (15)	15.0 (3)		
3 = severe redness, % (*n*)	0	5 (1)		
Lid margin redness, right eye			*0.162*	
0 = no redness, % (*n*)	0	0		
1 = mild redness, % (*n*)	69.4 (25)	45.0 (9)		
2 = moderate redness, % (*n*)	25.0 (9)	50.0 (10)		
3 = severe redness, % (*n*)	5.6 (2)	5.0 (1)		
Lid margin redness, left eye			*0.266*	
0 = no redness, % (*n*)	2.8 (1)	0		
1 = mild redness, % (*n*)	66.7 (24)	45.0 (9)		
2 = moderate redness, % (*n*)	25.0 (9)	50 (10)		
3 = severe redness, % (*n*)	5.6 (2)	5 (1)		
Conjunctival fluorescein staining, right eye			*0.108*	
0 = absent/very slight, % (*n*)	5.6 (2)	25 (5)		
1 = mild, % (*n*)	80.6 (29)	65 (13)		
2 = moderate, % (*n*)	13.9 (5)	10 (2)		
Conjunctival fluorescein staining, left eye			*0.207*	
0 = absent/very slight, % (*n*)	5.6 (2)	20 (4)		
1 = mild, % (*n*)	77.8 (28)	60 (12)		
2 = moderate, % (*n*)	16.7 (6)	20 (4)		
Cornea fluorescein staining, right eye			*0.503*	
0 = absent/very slight, % (*n*)	44.4 (16)	60 (12)		
1 = mild, % (*n*)	38.9 (14)	25 (5)		
2 = moderate, % (*n*)	16.7 (6)	15 (3)		
Cornea fluorescein staining, left eye			*0.656*	
0 = absent/very slight, % (*n*)	47.2 (17)	60 (12)		
1 = mild, % (*n*)	33.3 (12)	25 (5)		
2 = moderate, % (*n*)	19.4 (7)	15 (3)		

Results presented as measurement results (SD) or number (percentage). Italic indicates significant differences between groups at *p* ≤ 0.05

*SD*, standard deviation; *n*, number; *CO*, healthy control group; *AD*, Alzheimer’s disease group; *TBUT*, tear break-up time

## Results

### Demographic Data

The demographic data of the participants are presented in Table 1. MMSE results and the number of *ApoE* allele 4 carriers differed significantly between the study groups. The mean MMSE was 28.6(±1.4) in the CO group and 23.9(±2.8) in the AD group (*p* < 0.0001). The proportion of *ApoE* allele 4 carriers was 44.1% in the CO group and 82.4% in the AD group (*p* < 0.009).

### Ocular Data

TF production was measured in both eyes by the Schirmer test. The two study groups did not significantly differ in TF production, tear break-up time (TBUT), lid margin redness, conjunctival redness, or ocular surface staining (OSS) (Table 2).

### Proteomic Data

In total, we identified 47 proteins in TF samples that were up- or downregulated in the AD group. We classified the TF proteins into three groups based on their protein function. Two of the three function reports are already published (Kärkkäinen et al. 2024; Kärkkäinen et al. 2025). Here, we report 14 proteins that can be linked to inflammation and AD pathogenesis. The decrease or increase in protein amount (ratio) in AD samples was compared to the CO samples (normal = 1). For each, the protein amount was significantly (*p* ≤ 0.05) decreased (ratio < 0.5) or increased (ratio > 2.0) in the AD group (Figure 1). For label-free quantification using PEAKS, ID-directed label-free quantification was performed with outlier removal. The following parameters were applied regarding peptide features: quality ≥ 4, peptide ID count per group ≥ 1, and detected in ≥ 1 sample per group. The following parameters were applied to the proteins: FDR ≤ 5%, fold change ≥ 2, and significance on analysis of variance with ≥ 2 peptides. The significance score was calculated as the −10 log10 of the significance testing *p*-value. The average peak areas of all samples belonging to each group are compared to determine the ratio.

In order of the highest to lowest ratio, significantly upregulated proteins were SPARC-like protein 1 (SPAR1), immunoglobulin heavy variable 5–51 (HV551), immunoglobulin J chain (IGJ), serine/threonine protein phosphatase (CPPED1), mesothelin (MSLN), chitinase-3-like protein (CHI3L2), glucose-6-phosphate isomerase/neuroleukin (G6PI/NLK), annexin A3 (ANXA3), alpha-1-acid glycoprotein (ORM1), sialic acid synthase (SIAS), fibrinogen alpha chain (FGA), and alpha-1-antichymotrypsin (SERPINA3). Downregulated proteins were mammaglobin B (SCGB2 A1) and prolactin inducible protein (PIP). Figure 1 shows the CO:AD difference and up- and downregulated proteins. The dashed line represents a CO value of 1.

The figure shows up- and downregulated tear fluid (TF) proteins in participants classified as cognitively healthy controls (CO) and participants with Alzheimer’s disease (AD), given as ratio of CO to AD values (x-line) and significance score (y-line). CO value is 1 and marked in dashed line in figure. The differences between AD and CO were considered significant (*p* < 0.05) if the ratio value was <0.5 or >2.0.

## Discussion

The development of novel, easy-to-use detection methods and the discovery of new biomarker molecules for early AD are urgently needed. A potential novel source for biomarker molecules may be protein-rich TF, which has been reported to exhibit altered protein levels in several systemic diseases, including AD (Kalló et al. 2016; Kaštelan et al. 2023; Gijs et al. 2021). TF consists of a wide variety of proteins, which are important for maintaining the homeostasis of the ocular surface, and for the nutritional, immunological, and antimicrobial functions of the eyes (Perumal et al. 2015). Inflammation is known to be an early response in the AD brain and causes several changes in the function of cells (Kiraly et al. 2023; Liddelow et al. 2017; Pelkmans et al. 2024). In this study, we examined altered expressions of 14 TF proteins in the AD group compared to the CO group.

First, we aimed to eliminate factors that may affect TF protein levels. The AD and CO groups were homogenous in terms of demographic and eye data, and TF secretions, consistent with earlier findings (Kenny et al. 2019). During recruitment, we excluded the potential participants with eye diseases or diabetes, both of which may affect TF protein expression. The CO and AD groups differed only in their MMSE results, which were worse in the AD group, and the proportion of ApoE4 carriers, which was higher in the AD group. Participants in the AD group represented mild AD dementia based on their CDR interview; they all had a diagnosis for AD, and they had medication for AD. Participants in the CO group represented cognitively healthy.

PIP, LCN1, LYZ, IgA, SCGB2 A1, and MUC5 AC are common tear-specific proteins, which are responsible for the homeostasis of TF and functionality of the eye. Many TF proteins are produced in the lacrimal gland (Shigeyasu et al. 2016; Stoeckelhuber et al. 2006; Zhao et al. 2010). We found four altered TF proteins (SCGB2 A1, PIP, LCN1, and LYZ) produced by the lacrimal gland; interestingly, all were downregulated. SCGB2 A1 and PIP were significantly downregulated, while LCN1 and LYZ showed non-significant downregulation. Earlier studies have reported that LYZ, PIP, and LCN1 are downregulated in AD (Kenny et al. 2019; Kalló et al. 2016) and lacrimal gland dysfunction has been suggested, which may be the reason for the observed reduced expression in our study (Kalló et al. 2016).

PIP has important roles in the regulation of protein and enzyme activities, endocytosis, toll-like receptors, purinergic signalling, chemotaxis, and migration. In addition, PIP has been reported to play a crucial role in the regulation of microglial-mediated neuroinflammation, also linked to AD (Ernest James Phillips and Maguire, 2021; Jüngert et al. 2022). Prolactin is a pituitary gland hormone, also produced in the lacrimal gland. On the ocular surface, prolactin induces the expression of PIPs (Jüngert et al. 2022). According to an earlier study, prolactin levels are decreased in many neurodegenerative diseases (Ernest James Phillips and Maguire, 2021). PIP level is therefore considered a potential biomarker for both degenerative diseases of the eye and neurodegenerative diseases (Jüngert et al. 2022).

Our results concerning downregulated proteins were consistent with earlier reports, although some of these proteins were not significantly altered. The alteration was in the same direction as previously reported (Kenny et al. 2019; Kalló et al. 2016), and these changes may cause synergistic effects together with other poorly functioning proteins. The hypothesis of lacrimal gland dysfunction in AD may also contribute to the development of pathophysiological changes in the eyes of AD patients.

CPPED1 is reported to dephosphorylate AKT1 at Ser473, blocking the cell cycle and promoting apoptosis (Zhuo et al. 2013). CPPED1 is also involved in glucose uptake in adipose tissue and is suggested to mediate glucose metabolism via the PI3 K-AKT signalling pathway (Vaittinen et al. 2013). In our study, CPPED1 was upregulated in the AD group. Upregulation of CPPED1 may indicate increased apoptosis and disturbances in glucose metabolism, both of which may contribute to the progression of AD (Zhuo et al. 2013; Vaittinen et al. 2013).

In the early phase of Alzheimer’s disease (AD), microglia and astrocytes transit from their normal phenotypes to a reactive neuroinflammatory phenotype (Kiraly et al. 2023). In our study, we detected increased CHI3L2 protein levels in the AD group. CHI3L2 is known to be a fluid biomarker associated with the reactive form of glial cells, primarily indicating the presence of reactive astrocytes and, to a lesser extent, microglia (Sanfilippo et al. 2023). Our findings suggest that in mild AD dementia, a neuroinflammatory state may be developing, with astrocytes transitioning into their reactive form. CHI3L2 may represent a promising biomarker candidate, and TF could serve as a convenient alternate source for its detection, potentially replacing CSF.

FGA protein is a plasma factor produced by enzymatic cleavage during the coagulation cascade in response to bodily injury (Weisel 2005). It also accumulates in nerve cells and is an important regulator of the nervous system through its triggering of reactive astrogliosis, inhibition of neurite outgrowth, and promotion of vessel wall inflammation and vascular permeability in endothelial and smooth muscle (Bian et al. 2021). In the AD brain, the accumulation of FGA is associated with pathology in neural cells and blood vessels (Bian et al. 2021). Consistent with earlier reports, we observed upregulated FGA in the AD group (Bian et al. 2021). We speculate that FGA may also be a suitable biomarker candidate, indicating the presence of inflammation.

SPARCL1 belongs to the extracellular matrix protein family secreted by astrocytes. In the brain, SPARCL1 exists in the postsynaptic membrane of cells and has roles in synapse formation, maturation, pruning, and plasticity (Gan & Südhof 2020). After traumatic CNS injury, reactive astrocytes and SPARCL1 together have protective roles in tissue repair and synaptic reorganisation (Kim et al. 2021). In our study, SPARCL1 was upregulated in the AD group, which may indicate an increased demand for tissue repair and neuroprotection during the mild stages of AD dementia.

SERPINA3 is classified as a protease inhibitor and is produced in the liver but distributed throughout the body (Romero-Sevilla et al. 2022). In the brain, SERPINA3 is expressed in astrocytes that colocalise with Aß plaques and may therefore be linked to AD (Liu et al. 2021). SERPINA3 is speculated to have a role in cognitive decline, although the exact mechanism has not yet been clarified. SERPINA3 activity may increase the rate of amyloid fibril aggregation and phospho-tau formation and increase apoptosis of neuronal cells (Romero-Sevilla et al. 2022). SERPINA3 is also considered a potential biomarker, as it is reported to increase in people with amnestic mild cognitive impairment. SERPINA3 protein levels can be detected in serum and CSF (Romero-Sevilla et al. 2022: Liu et al. 2021). We detected increased SERPINA3 levels in the AD group, in line with previous findings (Romero-Sevilla et al. 2022: Liu et al. 2021). In addition, our results suggest that TF may be a source for the detection of SERPINA3 levels together with serum and CSF.

We also found changes in a protein specifically linked to the active state of microglia. ANXA3 belongs to calcium-dependent proteins, and its role in humans is poorly understood. ANXA3 is a microglia-specific marker thought to be expressed in the cytoplasm in both the resting and activated states; a study in rats suggests its expression is increased in the activated state. ANXA3 is speculated to increase the proliferation and migration of microglia (Zhang et al. 2021). ANXA1 is a better known annexin protein than ANXA3, associated with the pathogenesis of various neurodegenerative and neuroinflammatory diseases and may serve as a promising therapeutic target (de Souza Ferreira et al. 2025). Increased expression of ANXA3 in our AD group may indicate the presence of active microglia, but its use for therapeutic purposes needs further research.

ORM1 is a plasma glycoprotein associated with inflammation. It belongs to the acute phase proteins thought to modulate the permeability of microvessel walls and the blood-brain barrier. During trauma, levels of plasma ORM1 proteins are reported to increase (Yuan et al. 2010). In our study, the levels of ORM1 were increased in the AD group. We did not find an established link to AD in the literature, but the protein function fits well with the current understanding of AD pathology. High ORM1 levels may be associated with AD pathogenesis due to its properties of modulating permeability.

GPI is a multifunctional protein, whose name is dependent on its location either in the cytoplasm or extracellular phase. In the cytoplasm, the protein is called GPI and has a role in glycolysis and gluconeogenesis (Haga et al. 2006). Once present in the extracellular space, the protein’s function is neurotropic, and its name is NLK. In the literature, the role of extracellular NLK is associated with AD. NLK is expressed near to Aβ plaques and in Aβ-laden vessels in AD brains. Most AD patients are reported to have some degree of cerebral amyloid angiopathy, for which NLK is a potential biomarker. Increased levels of NLK can be detected in CSF in mild AD stages. High levels of NLK may therefore indicate both existing vascular amyloid depositions and cerebral amyloid angiopathy (De Kort et al. 2021). We detected increased GPI/NLK levels in the TF of our AD group. These findings may be associated with the process of Aβ plaque formation and cerebral amyloid angiopathy in mild AD dementia.

NANS is the enzyme that catalyses the posttranslational process, sialylation. Sialylation involves the attachment of sialic acid residues to target molecules, most often glycoproteins and glycolipids. It is important for the functionality of multiple molecules and processes, for example during nerve cell development, repair, and synapse formation, as well as during the regulation of immune response and invasion of pathogens (Fastenau et al. 2023; Yang et al. 2021). The amount of the catalysing enzyme NANS and sialic acid are rate-limiting factors of sialylation (Yang et al. 2021). The process is known to change throughout life and as the result of disease (Fastenau et al. 2023). The exact role of sialylation in the AD brain is poorly understood. Sialylation seems to have a link to AD because of its role in brain function, learning, and memory (Salminen & Kaarniranta 2009). In addition, Aβ plaques and amyloid precursor protein contain sialic acid residues (Fastenau et al. 2023). It is not surprising, then, that in our study levels of NANS were increased in the AD group. Increased levels of NANS may indicate a high amount of Aβ plaques and amyloid precursor protein in the brain.

MSLN is a cell surface glycoprotein, a so-called matrix protein, which is known to be vulnerable to oxidative stress and inflammation (Stepanov et al. 2022). Upregulated levels of MSLN are reported in patients with retinal vein occlusion (Stepanov et al. 2022). We report increased levels of MSLN in our AD group, which may indicate retinal changes or abnormalities associated with AD. In the literature, altered MSLN levels have not previously been associated with AD.

We also detected two upregulated plasma markers, IGHV5-51 and JCHAIN, in our AD group. Both of those proteins are markers of ongoing immune activation. IGHV5-51 is associated with coeliac disease (gluten activation), intestinal lesions, and infiltrated immune cells (Tutturen et al. 2018). As far as we are aware, IGHV5-51 has not been previously associated with AD. While AD is not typically associated with intestinal symptoms, recent studies have reported altered gut microbiome composition. These microbiome studies indicate that AD may also affect the intestine (Yu et al. 2024). Our finding may indicate the presence of inflammation in the intestines of the AD group.

JCHAIN is a small plasma polypeptide that regulates the formation of the important immunoglobulins IgA and IgM (Johansen et al. 2000). Secretory IgA is a common protein in TF (Shigeyasu et al. 2016). According to earlier studies, levels of immunoglobulins (including IgA) in the plasma are reported to correlate with the progression of AD, indicating their potential as plasma biomarkers for dementia (Yaqub et al. 2023). We detected increased levels of JCHAIN in the AD group; because JCHAIN is a regulator of IgA, this may indicate increased levels of IgA in AD, consistent with earlier findings. However, changes in IgA levels were not detected in the TF samples.

In this study, we report 14 TF proteins whose expression was altered in the study group, which represented mild AD dementia; the study participants all had an AD diagnosis and medication. According to our performed literature search, the protein functions may link inflammation or the body’s defence system (Kiraly et al. 2023; Liddelow et al. 2017; Pelkmans et al. 2024). Our goal for this study was to search for new potential biomarker candidate molecules to reveal the early stage of AD. These 14 proteins represent a potential biomarker candidate and can be used as reference proteins in our following studies investigating other study groups that represent more earlier stages of AD or other demented diseases.

The limiting factors of the study were the relatively small sample size in our pilot study. We conducted a pilot study first with the AD group to find out whether the TF proteins were altered or not and after that continued the recruitment of larger study groups. Moreover, we did not examine the levels of Aβ or tau proteins in our TF samples. All our observations in this study and our other studies (Kärkkäinen et al. 2024; Kärkkäinen et al. 2025) need to be confirmed with larger data, and the levels of Aβ or tau proteins in our TF samples should be analysed from a larger cohort too. Now, we can only speculate that TF samples from the AD group may have contained Aβ or tau proteins.

## Conclusion

Current treatments for AD can only slow down the progression of AD; if new and future drugs and other treatments can be initiated in the mild stage of AD or even earlier, it is beneficial for patients, their families, and our society. We urgently need specific and easy-to-detect biomarkers and the sources of them for help to recognise early AD from other demented diseases. This study provides new information about TF proteins whose altered expression may reflect underlying pathophysiology, especially relating to neuroinflammation, in AD or the continuum of dementia. New information about the pathophysiological changes at the cellular level is useful for many research purposes, such as searching for novel biomarker molecules for AD. This study encourages us to continue the studies for a searching a new biomarker molecule with a larger study cohort.

## Data Availability

No datasets were generated or analysed during the current study.
